# Machine Learning of Protein Interactions in Fungal Secretory Pathways

**DOI:** 10.1371/journal.pone.0159302

**Published:** 2016-07-21

**Authors:** Jana Kludas, Mikko Arvas, Sandra Castillo, Tiina Pakula, Merja Oja, Céline Brouard, Jussi Jäntti, Merja Penttilä, Juho Rousu

**Affiliations:** 1 Helsinki Institute for Information Technology HIIT, Department of Computer Science, Aalto University, Espoo, Finland; 2 VTT Technical Research Centre of Finland, Espoo, Finland; Harbin Institute of Technology Shenzhen Graduate School, CHINA

## Abstract

In this paper we apply machine learning methods for predicting protein interactions in fungal secretion pathways. We assume an inter-species transfer setting, where training data is obtained from a single species and the objective is to predict protein interactions in other, related species. In our methodology, we combine several state of the art machine learning approaches, namely, multiple kernel learning (MKL), pairwise kernels and kernelized structured output prediction in the supervised graph inference framework. For MKL, we apply recently proposed centered kernel alignment and *p*-norm path following approaches to integrate several feature sets describing the proteins, demonstrating improved performance. For graph inference, we apply input-output kernel regression (IOKR) in supervised and semi-supervised modes as well as output kernel trees (OK3). In our experiments simulating increasing genetic distance, Input-Output Kernel Regression proved to be the most robust prediction approach. We also show that the MKL approaches improve the predictions compared to uniform combination of the kernels. We evaluate the methods on the task of predicting protein-protein-interactions in the secretion pathways in fungi, *S.cerevisiae*, baker’s yeast, being the source, *T. reesei* being the target of the inter-species transfer learning. We identify completely novel candidate secretion proteins conserved in filamentous fungi. These proteins could contribute to their unique secretion capabilities.

## Introduction

Protein secretion is a fundamental cellular process that is required for transporting proteins into cellular compartments, the cell surface and the external space of the cell as well as for covalent modification i.e. disulphide bond formation and glycosylation of proteins. As can be expected from its central role, the protein secretion machinery is conserved in eukaryotes. Fundamental research to unravel its functioning has been carried out in the fungus *Saccharomyces cerevisiae* [1]. However, the baker’s yeast *S. cerevisiae* of the subphylum Saccharomycotina does not naturally secrete large amounts of proteins unlike the filamentous fungi of the subphylum Pezizomycotina. For example the Pezizomycotina *Trichoderma reesei* (*Hypocrea jecorina*) is able to secrete its native cellulase proteins with yields of over 100 g/l in industrial cultivations [2]. With their protein secretion capabilities Pezizomycotina are central to industrial biotechnology. However, compared to *S. cerevisiae* their protein secretion machinery has not been studied in detail.

Protein-protein interaction data is very useful in defining cellular functions of proteins. Protein-protein interaction (PPI) is a term that covers various possible interactions between pairs of proteins from stable physical interactions to functional associations. While in many cases the molecular function of a new protein can be determined by sequence similarity searches, the molecular function tells little about the cellular function the protein might be carrying out in given conditions. However, the actual high throughput experimental measurement of protein-protein interactions has been limited to the most studied organism such as *E. coli*, *S. cerevisiae* and *Homo sapiens*.

The availability of verified annotations on protein function and interactions especially outside model organisms is currently a major bottleneck. In May 2015 the Genomes OnLine Database (https://gold.jgi-psf.org/) contained entries for almost 60 000 organisms. The number of sequenced species is growing exponentially and most importantly improvements in sequencing techniques allow the assembly of genome sequences of uncultivable micro-organisms from metagenomics samples [3]. In parallel the version 10.0 of protein-protein interaction database STRING [4] contained 2031 organisms. STRING combines a number of data sources, i.e. genomic neighbourhood, gene fusion, species co-occurrence, gene co-expression, experimental protein-protein interaction data and text mining results to predict protein-protein interactions. Furthermore, all taxonomic groups of organisms typically contain 10–20 percentage of lineage restricted genes i.e. genes that are not found in other taxonomic lineages [5]. Outside model organisms the function of these genes is typically unknown. With sequencing of uncultivable micro-organisms this percentage is likely to increase.

To bridge this gap computational PPI prediction has been intensely studied in the last decade. Early approaches focused on inferring functional PPIs from genomic context such as gene neighbors, gene clusters, Rosetta stone, and phylogenetic profiles as well as protein sequence co-evolution as reviewed in [6]. Lately, the research field has developed methods for predicting PPI networks of physical interactions [7, 8], pathway memberships [7] and more general biological networks such as gene regulatory networks and metabolic networks [8, 9]. Commonly experimental data is used as input features such as microarray/co-expression [8, 9], sometimes also more high level features such as domain knowledge, phylogenetic profiles and interologs [7]. In [8] a good review on approaches for *de novo* as well as for supervised biological network inference is given.

Transfer of protein interactions based on sequence homology is a widely used technique, but requires strict amino acid sequence identity cut-offs, for example above 80 percent, to be reliable [10, 11]. This limits its use to lineages where not much sequence diversification has occurred. For example homologous genes between species belonging to the fungal genus Aspergilli, of the subphylum Pezizomycotina, have only 68 percent average amino acid identity [12]. Furthermore, recently duplicated and hence sequence wise similar genes often change their function i.e. neofunctionalise. When comparing duplicated genes between species it has been found that orthologous gene pairs are more likely to retain functions than paralogous (for review [13]). However, using the orthology-paralogy relationships in function transfer would require that they would be first solved. Although numerous methods exist for this, on genome scale this is still not a trivial task. For example, the commonly used best-bi-directional hit technique can easily be misled in multigene families.

To overcome the sequence similarity requirement of annotation transfer, machine learning methods have been developed for PPI prediction over the last decade. In particular, the supervised network inference paradigm [14] takes the PPI prediction as a binary classification problem, to predict, whether a pair of proteins interact or not. Thus, any general model for classification learning is applicable in this setting, including ensemble learners [15–17], Naive Bayes, and support vector machines (SVM). SVM models rely on so called pairwise kernels, where the similarities of protein pairs are compared to each other. Another class of PPI learning methods aim to predict interaction patterns by learning similarities between proteins in the protein interaction network. Output kernel trees [18] and input-output kernel regression [19] are recent examples of this kind of methods.

The above approaches have not been explicitly applied to cross-species transfer learning, perhaps due to the limited amount of verified PPIs in a majority of species. Beyond basic sequence comparisons, more advanced computational methods have been applied in the cross-species setting only sparingly. In [20], a cross-species cluster co-conservation method is proposed, that exploits phylogenetic profiles for predicting protein interaction networks. In [21], a link propagation approach was proposed relying on gene expression and sequence similarity, applied to cross-species metabolic network reconstruction.

There is a dire need for novel function and interaction prediction methods that would be locally available, able to cross large sequence similarity distances and not require the solving of orthology-paralogy relationships to cope with the rising amount of genomes. In this paper, we introduce a framework of machine learning methods that can be used for predicting physical or functional protein-protein interaction or more specific biological networks i.e. metabolic pathways depending on what type of training labels are used. Our method uses as features various sequence similarity and protein family analysis derived from the CoReCo pipeline [22]. Although our method relies partly on sequence similarity, it is, through a combination of methods, still able to predict for proteins that do not belong into any known protein family. Hence our method can give clues for PPIs of previously unknown proteins. Our method introduces recently proposed multiple kernel learning (MKL) methods [23] to supervised network inference, thus boosting the performance of the latter method family and making full use of the wide array of sequence-derived features.

We focus in predicting the secretion machinery in industrially relevant fungi, in particular, *T.reesei*. Our focus is in predicting functional protein-protein interactions (PPI) in the secretory pathway. As there are no verified protein interaction data available for these organisms, we assume the cross-species transfer learning setting, where the training data comes from *S. cerevisiae*, and prediction targets is *T.reesei*.

## Materials and Methods

### Data and preprocessing

#### Sequence data

In this paper the models are based on features that can be computational derived from protein sequence data. The sequence data for the two studied organisms were downloaded from SGD database (http://www.yeastgenome.org) for *S. cerevisiae* and from JGI Mycocosm database (http://genome.jgi.doe.gov/Trire2/Trire2.home.html) for *T. reesei*.

#### Protein-protein interaction data

The machine learning methods require a set of known PPIs to be used as ground truth for the model output, used for training and testing the model. We obtained our PPI data from the recently published genome-scale model of the yeast secretory machinery [24] that gathers knowledge of 50 years of research on secretion in *S.cerevisiae*. The authors identified 162 proteins to be involved in secretion that are assigned to 16 subsystems such as translocation, ER glycosylation, COP, Golgi processing etc. These protein complexes give 2200 undirected interactions between the 162 secretion proteins which are used as training labels.

#### Feature extraction

For our models we use several types of features to characterize the similarity of proteins as well as the similarity of protein pairs. For all protein sequences of the 2 organisms we computed the following features using the CoReCo pipeline [22]: sequence alignment with BLAST against the UniProt database as well as Global Trace Graph (GTG) [25], protein domains and functional sites gathered by InterProScan [26] from its member databases: Pfam [27], Panther [28], Gene3D [29], PRINTS [30], Prosite [31], PIRSF [32], SMART [33], and SUPERFAMILY [34] (See S1 Table for details on these data sources).

#### Artificial sequences

**Fig 1:**
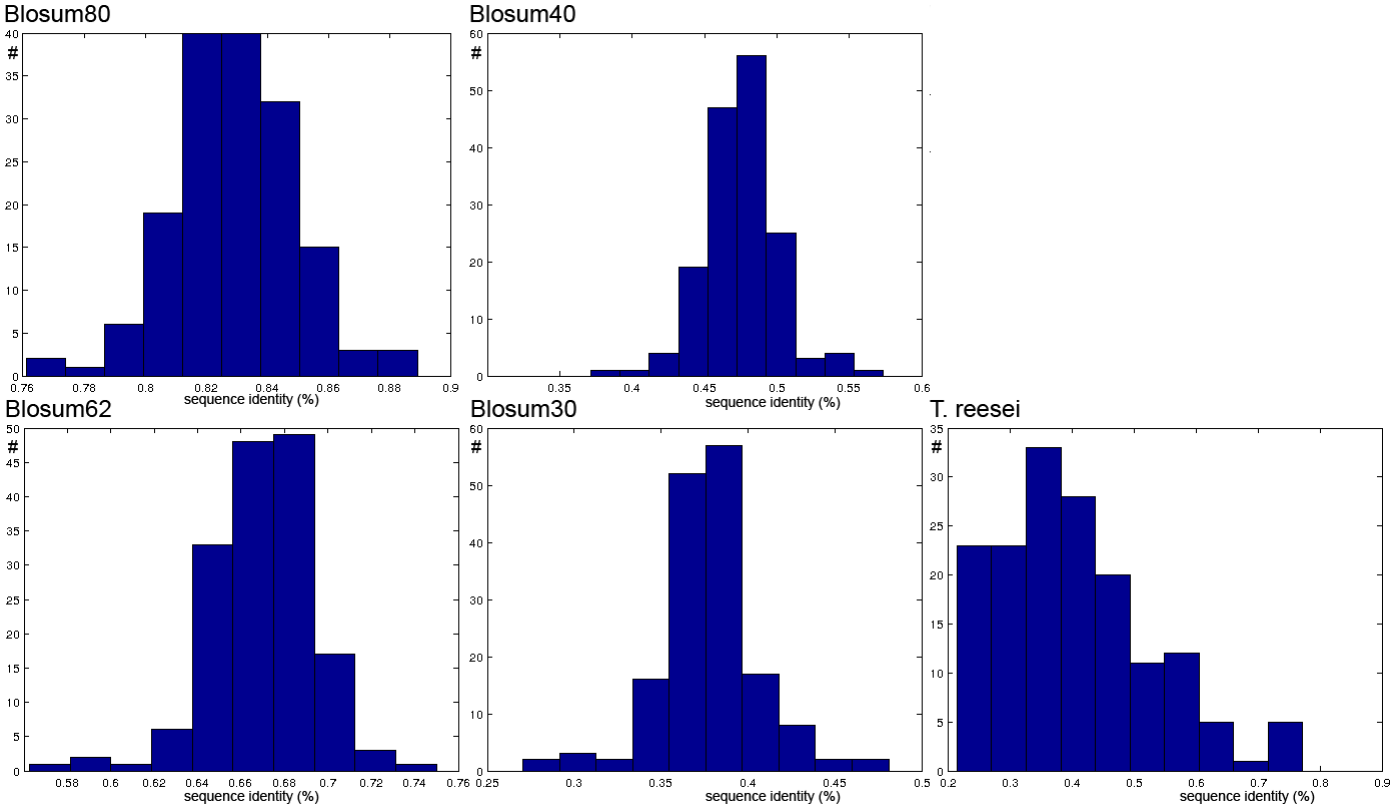
Frequency distribution of percentage of amino acid sequence identity between natural *S. cerevisiae* sequences and (1) sets of artificial sequences created from from *S. cerevisiae* with different Blosum matrices, (2) natural *T. reesei* sequences.

We used artificial data to test if the different biological network inference algorithm that have been developed for intra-species prediction also work for inter-species prediction with low sequence similarity. They are well below commonly used amino acid sequence identity cut-off values. For obtaining artificial sequences with varying levels of sequence similarity we altered the sequences of the 162 secretion proteins of *S. cerevisiae* based on Blosum matrices [35]. These matrices represent the substitution probabilities from an amino acid to an other amino acid in natural sequence data sets. Hence, they allow approximation of natural sequence evolution. We created four different data sets where we deleted and mutated 70%, 60%, 38% and 20% of the amino acids according to the Blosum30, Blosum40, Blosum62 and Blosum80 respectively. The Blosum matrices were downloaded from NCBI Blast site (ftp://ftp.ncbi.nih.gov/blast/matrices/). Each different Blosum matrix has been made by combining proteins that are no more similar than a given percentage (30%, 40%, 62% and 80%) to one single sequence and then comparing only those sequences [35]. In Fig 1, the percentage of amino acid sequence identity between the artificially mutated protein sequences of *S. cerevisiae* and *T. reesei* based on the Smith-Waterman alignment is shown. Based on visual comparison, the generated Blosum30 data set has a similar level of sequence similarity to *S. cerevisiae* as *T. reesei*. In the experiments, the artificially perturbed sequences were coupled with the labels of the corresponding labels of the original sequences.

#### Transcriptomic data analysis for biological network validation

The transcriptomic data for the validation of *T. reesei* PPI network was composed by eight publicly available data sets taken from Gene expression omnibus [36] plus eight in-house data sets. The public data sets contained 76 samples all together and the in-house data sets 499 samples. Once combined the final data set contained 575 samples and 9078 genes. Each data set was normalized separately using quantile normalization [37] and normalized again after they were combined using COMBAT normalization [38].

### Problem formalization

Supervised graph inference has been introduced a decade ago in [14] and has been widely used for biological network reconstruction subsequently. Given a set of nodes *V* = *v*_1_, .. ,*v*_*m*_ a biological network can be defined as an undirected graph *G* = (*V*, *E*) where *E* ⊂ *V* × *V* are the edges between the *m* vertices. The graph can be represented by a symmetric adjacency matrix *Y* = (*y*_*ij*_) of size *m* × *m* where *y*_*ij*_ = *y*_*ji*_ = 1 if the nodes *v*^*i*^ and *v*^*j*^ are connected and *y*_*ij*_ = *y*_*ji*_ = 0 otherwise. We will also use the shorthand $y\left(v_{i}\right)={\left(y_{ij}\right)}_{j=1}^{m}$ to denote the connectivity pattern of protein *v*_*i*_ in the network. In addition, we assume that each node has assigned features *x*(*v*_*i*_) ∈ *χ*, for some input space *χ*.

The learning task is then defined as follows: given partial knowledge of the graph *G* = (*V*, *E*) and the feature representation of the nodes, determine a function *f*: *V* × *V* → {0, 1} that best approximates the unknown edges of the graph.

**Fig 2:**
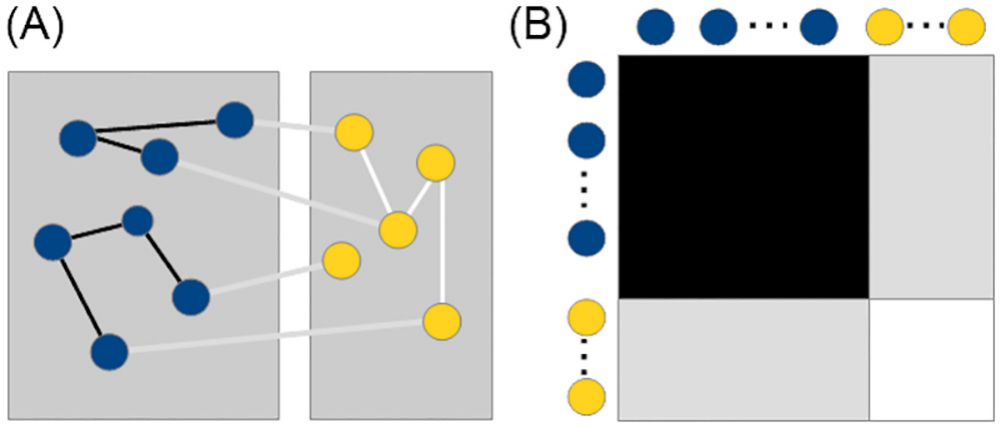
Schematic representation of the duality between (A) the PPI network and (B) the adjacency matrix for the proteins in the training set (blue) and testing set (yellow) and their interactions: training interactions (black), training-testing interactions (gray) and testing interactions (white).

Note that the main difficulty for solving this problem is that the features are assigned to individual nodes and the labels to pairs of nodes [9]. To transform the task into a standard classification problem, we use a global approach that tries to find a feature representation for pairs of nodes. Another issue inherent to biological network inference is the substantial class imbalance since the number of positive interactions is small compared to the number of all possible interactions. Thus special care is needed for setting up the evaluation experiments, see e.g. [39]. First of all, the evaluation metrics should be chosen such that the class imbalance does not lead to incorrect conclusions (e.g AUPR metric explained below). Secondly, methods that predict for each protein an interaction profile (see OK3 and IOKR below), represented as a multilabel, a binary vector containing interaction labels for all other proteins, are able to mitigate the class imbalance, since in general the set of multilabels are diverse with no very frequent multilabel. In [9] it is recommended to perform cross validation on the nodes as cross validation on pairs tends to give too optimistic results. A schematic representation of the duality between the biological network and the adjacency matrix and the cross validation on nodes is given in Fig 2.

Finally, for performing inter-species biological network inference we use the protein sequences and their interactions from one species as training set and the protein sequences from the second species as testing set. Note that in this setting the training-testing interactions are not of interest and that the feature representation needs to be the same for training and testing proteins.

### Inference Algorithms

In this section we present three different approaches for supervised network inference that we have applied to inter-species PPI network prediction. Additionally, we present different approaches for learning kernels that account for the relevance of a data source for the learning task.

#### Output kernel trees (OK3)

have been proposed by [18] and are based on the kernel embedding of the graph where the kernel function is defined as *k*_*Y*_: *V* × *V* → ℜ with *k*_*Y*_(*v*, *v*′) = 〈*ψ*(*v*), *ψ*(*v*′)〉. The kernel *k*_*Y*_(*v*, *v*′) is defined such that adjacent vertices have higher values of *k*_*Y*_ than non-adjacent ones. To achieve this, the diffusion kernel is commonly used *K*_*Y*_ = exp(−*βL*) where *L* is the Laplacian matrix of the graph *L* = *D* − *Y* with *D* being the degree matrix and *Y* the adjacency matrix. Additionally, *β* > 0 is a user defined parameter that controls the diffusion degree.

The OK3 algorithm relies on the top-down induction algorithm widely used to learning decision trees (e.g. CART [40]). The methods start with a tree represented by a single leaf and then recursively partition (or split) the input data *S* until the data is homogeneous enough (in our case: the proteins in *S* have similar connectivity patterns). The data arriving to leaf *L* of the decision tree is split into two parts *S*_*l*_ and *S*_*r*_, using a binary test *T*_*t*_(*x*) ∈ {0, 1} based on a value of a single input feature of *x* (e.g. does protein have a given motif or not). The two sets *S*_*l*_ = {*x* ∈ *S*|*T*(*x*) = 0} and *S*_*r*_ = {*x* ∈ *S*|*T*(*x*) = 1} will be recursively used to grow subtrees which then will be attached as the children of *L*.

For learning the decision trees on the input vectors *x*_*i*_ = *x*(*v*_*i*_), *i* = 1..*m* the following score is maximized to select a test *T* to be inserted in the decision tree leaf given the set of inputs *S* routed to the current decision tree leaf:

$$\begin{array}{c} Score\left(T,S\right)=var\left\{\psi \left(v\right)|S\right\}-\frac{N_{l}}{N}var\left\{\psi \left(v\right)|S_{l}\right\}-\frac{N_{r}}{N}var\left\{\psi \left(v\right)|S_{r}\right\} \end{array}$$ (1)

where *ψ*(*v*) is the output feature vector, *N*, *N*_*l*_ and *N*_*r*_ are the sizes of the training sample *S* and its left and right split, *S*_*l*_ and *S*_*r*_, respectively. The variance of the output feature vectors in the set *S* can be easily computed using the kernel trick:

$$\begin{array}{c} var\left\{\psi \left(v\right)|S\right\}=\frac{1}{N}\sum_{i=1}^{N}k_{Y}\left(v_{i},v_{i}\right)-\frac{1}{N^{2}}\sum_{i,j=1}^{N}k_{Y}\left(v_{i},v_{j}\right) \end{array}$$

One main advantage of the OK3 approach is that the decision tree on the input features results in a ranking of relevant features for the learning task.

Then for prediction each leaf *L* is labeled with a prediction ${\hat{\psi }}_{L}=\frac{1}{N_{l}}\sum_{i=1}^{N_{L}}\psi \left(v_{i}\right)$ analog to standard regression trees where *N*_*L*_ are the number of samples that reach the leaf. Finally, the kernel value between two vertices *v* and *v*′ where *x*(*v*) reaches leaf *L*_1_ and *x*(*v*′) leaf *L*_2_ respectively can be approximated by thresholding

$$\begin{array}{c} {\hat{k}}_{Y}\left(v,v^{\prime }\right)=\frac{1}{N_{L_{1}}N_{L_{2}}}\sum_{i=1}^{N_{L_{1}}}\sum_{j=1}^{N_{L_{2}}}k_{Y}\left(v_{i}^{1},v_{j}^{2}\right) \end{array}$$

where $v_{i}^{k},i=1,\ldots ,N_{L_{k}}$ enumerate the vertices routed to leaf *L*_*k*_. For improving the accuracy of the method an ensemble of decision trees also known as a random forest is used. In our experiments we used the C code provided by the authors [18].

#### Kernels on protein pairs

The main idea of the biological network reconstruction methods presented in [8] is to reformulate the task as a pattern recognition problem: given a training set *τ* = {(*u*_1_, *t*_1_),(*u*_2_, *t*_2_), .. ,(*u*_*N*_, *t*_*N*_)} of patterns *u*_*i*_ ∈ ℜ^*q*^ with a binary label *t*_*i*_ ∈ {−1, 1} infer a function *f*: ℜ^*q*^ → {−1, 1} for any new pattern *u*. The main hindrance in doing so is that in network reconstruction the labels are defined on pairs of vertices and the input features or patterns on individual vertices. Thus in a first step a so called linear kernel on pairs of vertices induced by their input features is defined by their inner product *k*_*X*_(*v*, *v*′) = *x*(*v*)^*T*^ *x*(*v*′). These kernels *k*_*X*_ represent the similarity of any pair of protein sequences that are then used to compute kernels on pairs of protein pairs as follows

1. Direct product kernel: *k*_*DRCT*_((*a*, *b*), (*c*, *d*)) = *k*_*X*_(*a*, *c*) * *k*_*X*_(*b*, *d*)
2. Tensor product pairwise kernel: *k*_*TPPK*_((*a*, *b*), (*c*, *d*)) = *k*_*X*_(*a*, *c*) * *k*_*X*_(*b*, *d*) + *k*_*X*_(*a*, *d*) * *k*_*X*_(*b*, *c*)
3. Metric learning pairwise kernel: *k*_*MLPK*_((*a*, *b*), (*c*, *d*)) = (*k*_*X*_(*a*, *c*) − *k*_*X*_(*a*, *d*) − *k*_*X*_(*b*, *c*) + *k*_*X*_(*b*, *d*))^2^

Now a standard support vector machine (SVM) can be used to solve the binary classification task. Since PPI networks are undirected the tensor product kernel *k*_*TPPK*_ and the metric learning pairwise kernel *k*_*MLPK*_ are best suited for modelling the similarity between protein pairs.

Despite the method’s good predictive performance it has a major drawback: the kernels between pairs of proteins can become quickly very large even for a reasonable amount of protein sequences. The space complexity for storing the kernel matrix turns out to be *O*(*m*^4^) where *m* is the number of proteins in the biological network which leads to serious scalability problems and usage of computational resources [21].

#### Input-Output Kernel Regression (IOKR)

This method combines elements of the two previous algorithms that circumvent their respective disadvantages—on the input side it uses the simple kernels on protein pairs and on the output side it uses the diffusion kernel built from the adjacency matrix of the output graph. But the classification problem is addressed by solving a kernel learning problem using regularized regression [19, 41]. The method comes in two flavors: the supervised version learns only the kernel ridge regression model and the semi supervised one adds a smoothness constraint using the inputs of labeled data and auxiliary data, called unlabeled data.

As the OK3 method, IOKR proposes to solve the link prediction problem by learning an output kernel $k_{Y}:V\times V\to R$, that encodes the similarities between the proteins in the interaction network. After learning this kernel, positive interactions can be predicted for the kernel values that are higher than some threshold *θ*:

$$\begin{array}{c} f_{\theta }\left(v,v^{\prime }\right)=sgn\left({\hat{k}}_{Y}\left(v,v^{\prime }\right)-\theta \right) \end{array}$$

As *k*_*Y*_ is a kernel, its values can be written as: *k*_*Y*_(*v*, *v*′) = 〈*ψ*(*v*), *ψ*(*v*′)〉, where *ψ* is called the output feature map. The IOKR method approximates the output feature map *ψ* with a function *h* and then build an approximation of the output kernel *k*_*Y*_ by taking the inner product between the values of this function:

$$\begin{array}{c} {\hat{k}}_{Y}\left(v,v^{\prime }\right)=\langle h\left(v\right),h\left(v^{\prime }\right)\rangle \end{array}.$$

Thus learning *f*_*θ*_ reduces to learn the single variable function *h*.

Then given models of the general form *h*_*M*_(*v*) = *Mϕ*(*v*) and assuming a regularized square loss function the parameters of the supervised IOKR model can be estimated based on *l* training samples as follows:

$$\begin{array}{c} {\text{argmin}}_{M}\sum_{1}^{l}∥h_{M}\left(v_{i}\right)-\psi \left(v_{i}\right){∥}^{2}+\lambda_{1}∥M{∥}_{F}^{2} \end{array}$$

where *λ*_1_ > 0 is a regularization parameter that is tuned with cross validation for the experiments.

The method has also been extended to the semi-supervised setting where the input of unlabeled data is taken into account. The new cost function that has to be minimized is:

$$\begin{array}{c} {\text{argmin}}_{M}\sum_{1}^{l}∥h_{M}\left(v_{i}\right)-\psi \left(v_{i}\right){∥}^{2}+\lambda_{1}∥M{∥}_{F}^{2}+\lambda_{2}trace\left(h_{M}L_{X_{n}}h_{M}^{T}\right) \end{array}$$

where *L*_*X*_*n*__ = exp(−*β*(*D*_*n*_ − *K*_*X*_*n*__)) denotes the diffusion kernel associated to input kernel matrix on labeled and unlabeled data. The last term constrains proteins that are similar to each other in input to be similar in the predicted interaction network. *λ*_1_ > 0 and *λ*_2_ > 0 are two regularization parameters that are tuned with cross validation for the experiments. Both minimisation problems lead to a closed form solution that can be found in Propositions 4 and 6 of [19].

#### Multiple Kernel Learning (MKL)

The heterogeneous set of features that we extracted from the protein sequences is expected not to uniformly contribute information to the learned model which makes the uniform combination of the kernels over the different data sources suboptimal. Therefore we apply Multiple Kernel Learning (MKL) to take the feature’s relevance into account. We focus on linear mixtures of kernels,

$$\begin{array}{c} K_{\mu }=\sum_{q=1}^{r}\mu_{q}K_{q} \end{array}$$

where the weights *μ*_*q*_ are typically restricted to be non-negative to ensure the PSD property of the resulting mixture. Note that setting *μ*_*q*_ = 1 for all kernels yields the uniform kernel combination. A major step forward in the MKL field was learning kernels based on centered kernel-target alignment [23]

$$\begin{array}{c} \hat{\rho }\left(K,K_{Y}\right)=\frac{{\langle K_{c},K_{Y}\rangle }_{F}}{∥K_{c}{∥}_{F}∥K_{Y}{∥}_{F}} \end{array}$$

where 〈.〉_*F*_ is the Frobenius product, ∥.∥_*F*_ the Frobenius norm, **K**_*Y*_ is a target kernel and **K**_*c*_ denotes a centered version of the input kernel *K*, achieved by the centering operation

$$\begin{array}{c} K_{c}=\left[I-\frac{11^{T}}{m}\right]K\left[I-\frac{11^{T}}{m}\right] \end{array}$$

where **1** denotes the vector of ones and **I** is the identity matrix.

This gives a simple improvement over the uniform combination of kernels be directly using the kernel-target alignment scores $\hat{\rho }\left(K_{q},K_{Y}\right)$ as a mixture weights:

$$\begin{array}{c} K_{\mu }\propto \sum_{k=1}^{p}\hat{\rho }\left(K_{k},K_{Y}\right)K_{k} \end{array}$$

This MKL method is called ALIGN. In [23] it is claimed that the kernel centering is critical for the kernel alignment score to correlate well with performance.

The previously presented independent kernel alignment neglects the correlation between the base kernels which can be overcome by jointly maximization the alignment between the convex combination kernel with the target kernel and is also referred to as ALIGNF:

$$\begin{array}{c} \max_{\mu }\frac{{\langle K_{\mu },K_{Y}\rangle }_{F}}{∥K_{\mu }{∥}_{F}} \end{array}$$

With the constraints that ‖***μ***‖_2_ = 1 and ***μ*** ≥ 0 the alignment maximization problem can be rewritten as:

$$\begin{array}{c} \mu^{*}={\text{argmax}}_{\mu }\frac{\mu^{T}aa^{T}\mu }{\mu^{T}M\mu } \end{array}$$

where **a** = (〈**K**_1*c*_, **K**_*Y*_〉_*F*_, …, 〈**K**_*rc*_, **K**_*Y*_〉_*F*_)^*T*^ records the kernel-target alignments of the input kernels and **M** = (*M*_*ql*_)_*ql*_ with *M*_*ql*_ = 〈**K**_*qc*_,**K**_*lc*_〉_*F*_ contains the pairwise kernel alignments between the input kernels. The problem can be solved by quadratic programming [23].

Another approach for optimizing the kernel target alignment has been proposed in [42]. The method aims at sparse combinations of kernels by regularizing the kernel weights by ℓ_*p*_-norm, where 1 ≥ *p* is simultaneously optimized. The proposed generalized ℓ_*p*_-norm kernel target alignment formulation is as follows:

$$\begin{array}{c} \min_{\mu \geq 0}\lambda_{1}{∥\mu -\mu_{0}∥}_{2}^{2}+\lambda_{2}\sum_{i=1}^{r}\mu_{i}^{p}-\sum_{i=1}^{r}\mu_{i}a \end{array}$$

The squared Euclidean distance in the first term is an instantiation of Bregman divergence [42]

$$\begin{array}{c} {\bar{B}}_{F}\left(\mu \right)=F\left(\mu \right)-{\left(\mu -\mu_{0}\right)}^{T}\nabla F\left(\mu_{0}\right) \end{array}$$

for *F*(***μ***) = 〈***μ***, ***μ***〉, and ***μ***_0_ is a fixed point in the domain of *F* (Following [42] we used ***μ***_0_ = 0 in our experiments.). Additionally, *λ*_1_ ≤ 0 and *λ*_2_ ≤ 0 are the regularization parameters. For implementing the sparsity inducing *l*_*p*_ regularizer *p* is systematically reduced towards unity till a sufficient level of sparsity is obtained. The solution of the path following is computed with a Predictor-Corrector algorithm [42].

### Evaluation metrics for Binary Predictions

Binary classification problems are typically evaluated with the accuracy measure which is computed as the number of correctly predicted pairs divided by the total number of pairs. For highly imbalanced problems like network inference accuracy is not an appropriate measure because it favours the majority class and thus the non-interactions. In the following Receiver-Operator-Characteristic (ROC) and Precision-Recall (PR) curves are presented which are better suited for evaluating network inference predictions [43].

Both measures are based on a so called confusion matrix which is 2 x 2 for binary classification with the columns and rows representing the predicted and the actual classes respectively. Denoting interactions as positive and non-interactions as negative the confusion matrix is given in Table 1.

**Table 1:** Confusion matrix indicating True positive (TP), False positive (FP), False negative (FN) and True negative predictions.

	Ground truth	
	P—Positive	N—Negative
Predicted Positive	TP—True Positive	FP—False Positive
Predicted negative	FN—false negative	TN—true negative

From this matrix several measures for model evaluation can be derived:

- True positive rate (TPR): also known as sensitivity or recall, is the number of true positives divided the number of the actual positives *TP*/*P*
- True negative rate (TNR): also known as specificity, is the number of true negatives divided by the number of actual negatives *TN*/*N*
- False positive rate (FPR): is the number of false positives divided by the number of actual negatives *FP*/*N*
- False negative rate (FNR): is the number of false negatives divided by the number of actual positives *FN*/*P*
- Precision: is the number of true positives divided by the number of predicted positives *TP*/(*TP* + *FP*)

All of these measures need to be combined in order to give a reliable performance measure of an algorithm e.g. specificity and sensitivity or precision and recall. Note as well that a threshold needs to be defined if predictions are confidence scores. For evaluating algorithms with varying confidence thresholds ROC and PR curves can be used.

#### ROC curves

plot the TPR over the FPR for varying confidence thresholds. More specifically, each threshold corresponds to a different confusion matrix and thus a different pair of values for TPR and FPR and a point on the ROC curve. The end points are always (0, 0) and (1, 1) and a perfect classifier would pass through the point (0, 1), while a random classifier would be a diagonal connecting (0, 0) and (1, 1). A common summary statistic of the ROC curve is the area under the ROC curve (AUROC). AUROC is one for a perfect classifier and 0.5 for a random one. For the highly imbalanced network prediction tasks even moderate FPR can lead to more FP predictions than TP predictions and hence a very low precision.

#### PR curves

plot the precision over the recall for varying confidence thresholds. The curve starts at a pseudo point (0, 1) and ends at (1, *P*/(*P* + *N*)) which corresponds to to predicting all pairs as positive. An optimal classifier would pass as well through (1, 1). The area under the PR curve (AUPR) is also a common summary statistic. As for AUROC one assumes that the higher the AUPR the better the performance of the method. One advantage of PR curves over ROC curves is that they allow to measure early precision where recall is low and thus gives a tool to evaluate the quality of the top ranks of the result list.

## Results

We report here on three sets of experiments. First, we evaluate how the prediction methods perform under simulated sequence data, representing differing amount of genetic distance between the source and target species. Second, we check how well the methods separate the secretory pathway from the rest of the genome. Third, we evaluate the PPI prediction in the cross-species transfer learning from *S. cerevisiae* to *T. reesei*.

### Network reconstruction for evolutionary distant sequences

Here we compare the performance of the network inference methods Output Kernel Trees (OK3), Tensor kernel SVM on protein pairs (PP), and supervised and semi-supervised Input-Output Kernel Regression (IOKR) for evolutionary distant species. As training data, we use the *S. cerevisiae* secretory pathway protein sequences as input and their functional interactions as labels. Then we try to predict these interactions in secretory pathway protein sequences that were perturbed using different BLOSUM matrices that correspond to different genetic distances.

**Fig 3:**
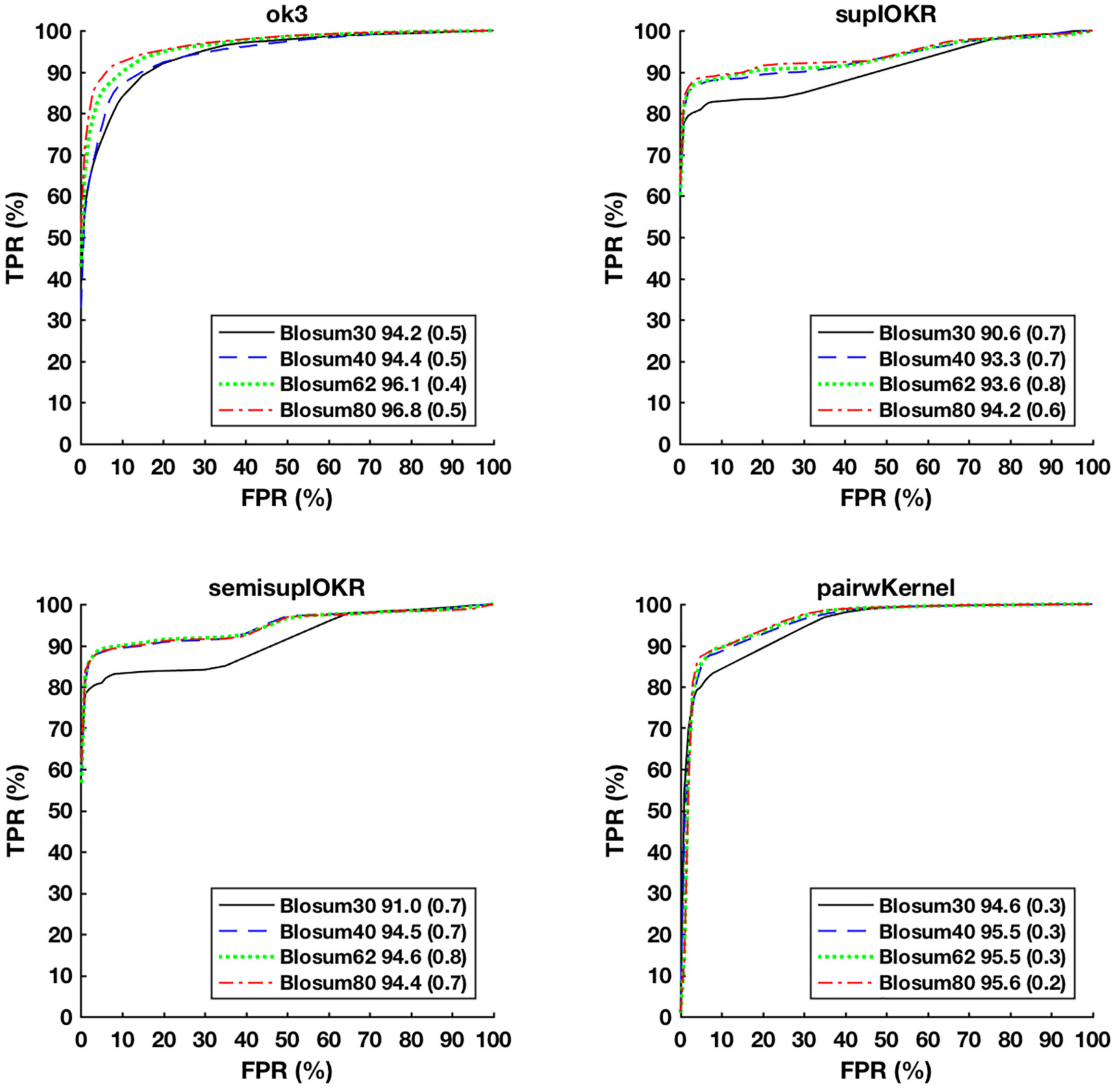
ROC curves for predicting PPIs in different artificial data sets with Output Kernel trees (OK3), Tensor kernels on protein pairs (Tensor Kernel on PP), and supervised and semi-supervised Input-Output Kernel Regression (IOKR). AUROC statistic of the associated curve is depicted in the figure legend (standard deviation in parenthesis).

Fig 3 depicts the Receiver operating characteristic curves (ROC) with associated area-under-curve (AUC) statistics for each inference method for the different evolutionary distances. As expected, all methods predict the better the smaller the distance with BLOSUM80 curves having the highest AUC and being closest to the top-left corner of the plots. The curves are averages of 20-fold cross-validation experiment.

In terms of AUC, OK3 obtains the best results, tensor kernel (PP) the second best and the IOKR methods being somewhat less accurate. However, closer examination of the method’s prediction performance for top-ranked interactions (*FPR* < 0.1) reveals that the IOKR methods in fact have the best early precision, thus would get the top-ranked interactions more accurately predicted than the competing methods.

The AUC statistics and the ROC curves of OK3 follow a smoothly worsening pattern with respect to the increasing evolutionary distance, while the other methods manifest a step change so that BLOSUM30 is markedly worse in AUC and lies clearly below the other curves.

**Fig 4:**
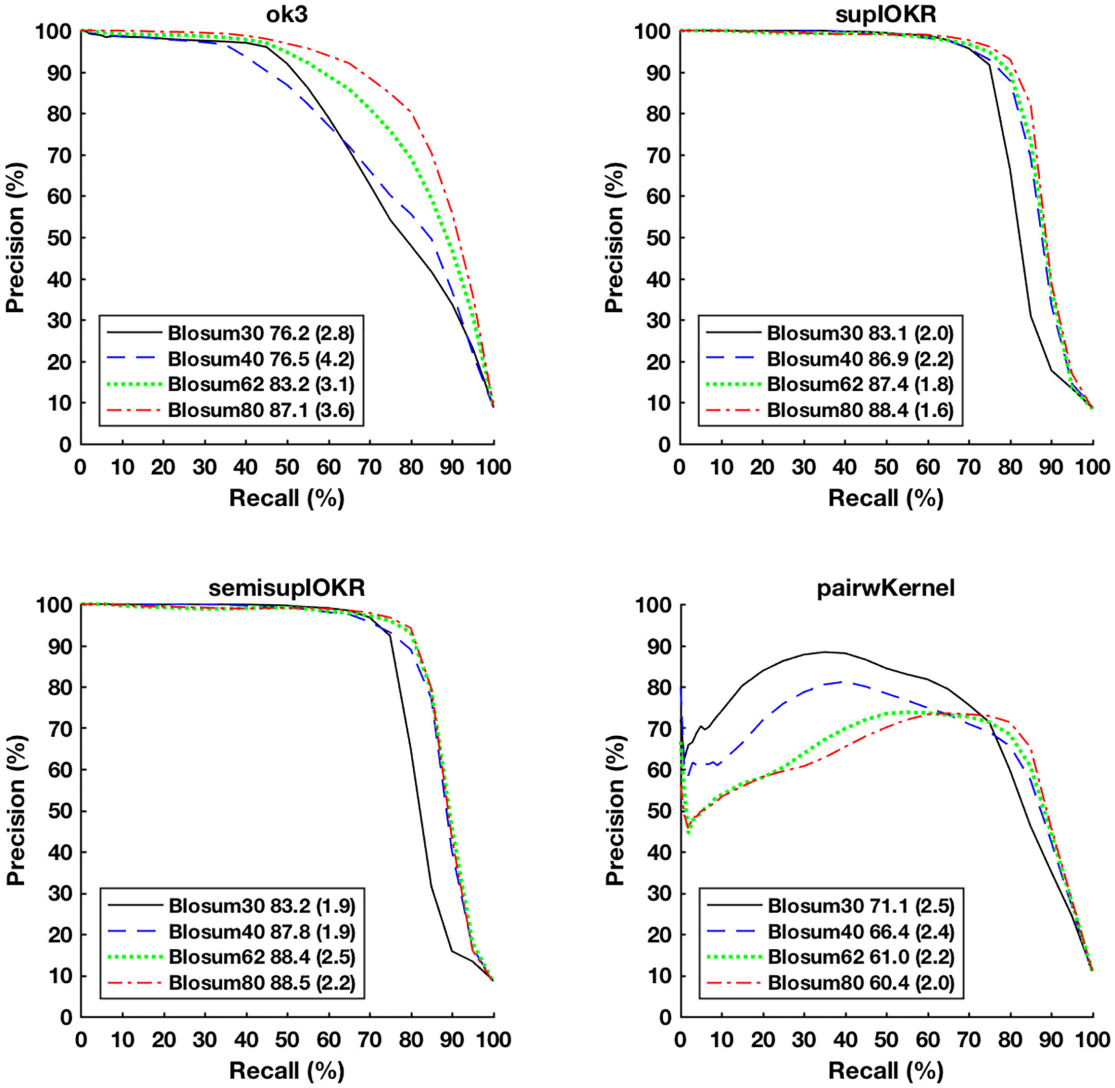
Precision-Recall (PR) curves for predicting PPIs in different artificial data sets with Output Kernel trees (OK3), Tensor kernels on protein pairs (Tensor Kernel on PP), and supervised and semisupervised Input-Output Kernel Regression (IOKR). AUPR statistic is shown in the legend for each curve (standard devation in parenthesis.

Fig 4 depicts the Precision-Recall (PR) curves of the same experiment. Here, the IOKR methods clearly perform best, having close to perfect precision regardless of the evolutionary distance until recall level of 0.5 and then a sharp drop at recall levels of 0.7–0.9 depending on the evolutionary distance. In contrast, OK3 manifests a close to one precision only for BLOSUM80 and for recall levels up to 0.3. Pairwise kernels do not obtain a high precision and produces a pattern that is inverted with respect to the evolutionary distance, indicating a high number of false positives in the SVM classifier and possible overfitting when the evolutionary distance is small.

The ROC and PR curves together indicate IOKR as the best compromise, given that both a high overall accuracy and high initial precision are desirable for network reconstruction.

### Identifying secretory pathway PPIs from full genome

Next, we check how well transfer learning of the secretory pathway works in the basic case of the source and target species being the same. In this experiment, the inference models were trained on the *S. cerevisiae* secretion proteins and their functional interactions, and the goal is to test the ability of the models to correctly identify the secretion pathway proteins among all *S. cerevisiae* proteins. In this setup, the ground truth is composed of PPIs between two secretory pathway proteins as the positive class and all other interactions as the negative class (true interactions between one or two non-secretory proteins as well as missing interactions between pairs of secretory pathway proteins).

**Fig 5:**
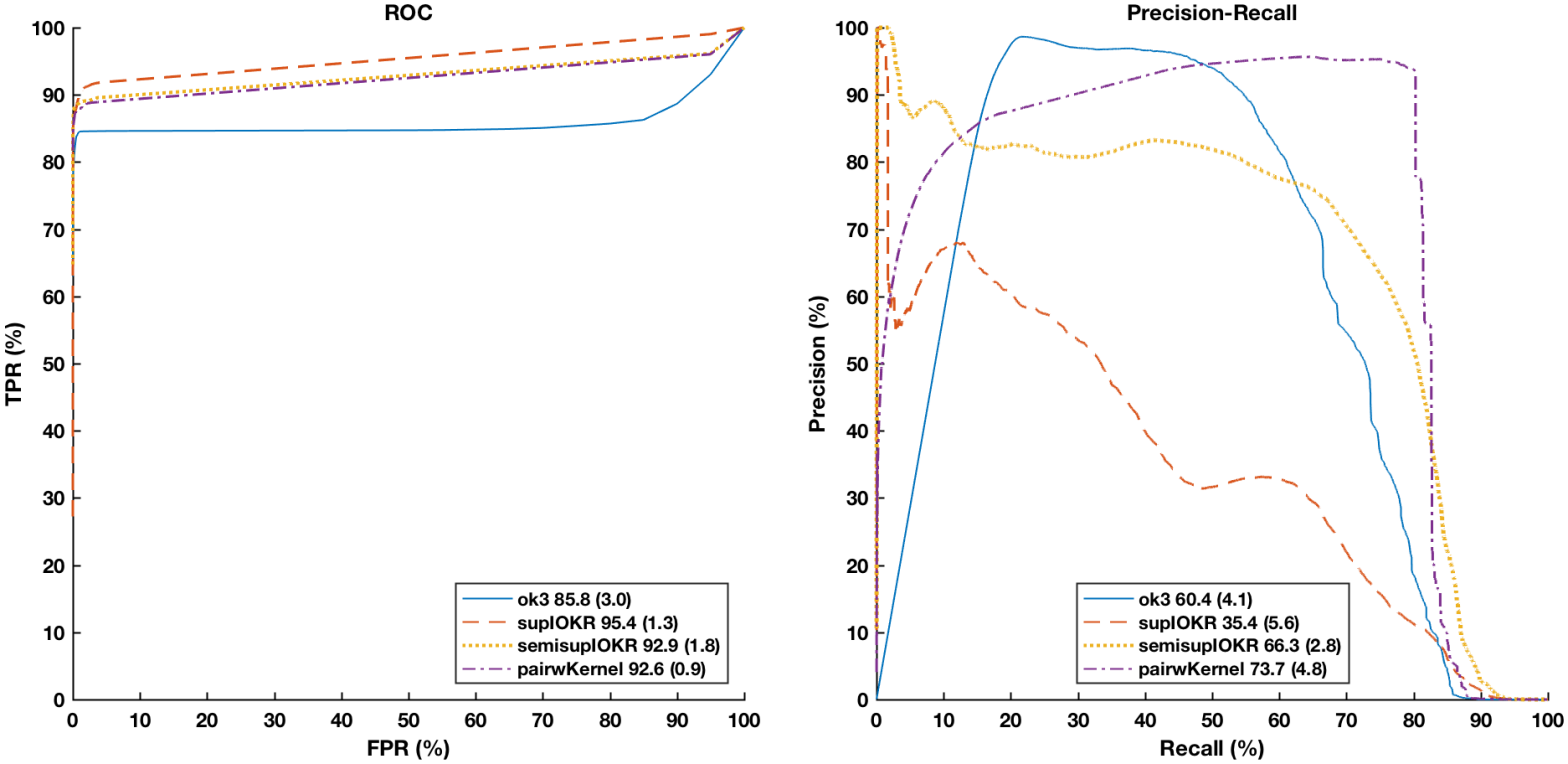
ROC curves and Precision-Recall (PR) curves for predicting secretory PPIs from the full *S. cerevisiae* genome with Output Kernel trees (OK3), Tensor kernels on protein pairs (Tensor Kernel on PP), and supervised and semi-supervised Input-Output Kernel Regression (IOKR). AUCROC and AUPR statistics are shown in the legend for each curve.

Fig 5 depicts the results of a 5-fold cross-validation experiment for the different network inference methods. In the ROC space (left pane), Pairwise kernels and the two IOKR methods are close in performance, with the semi-supervised IOKR being marginally better than the two others. OK3, however, performs significantly worse than the other three methods. In the precision-recall space (right pane), the two IOKR methods are the most robust in the low-recall regime, with the semi-supervised variant maintaining 0.7 precision rate up to 0.6 recall rate. Pairwise kernels and OK3 demonstrate a different pattern: they suffer from a high false positive rate in the low-recall regime, but have a good precision in mid-recall regime, before tailing off.

Analyzing the ROC and PR results together, semi-supervised IOKR emerges as the best compromise, due to its good ROC behaviour and good precision in the low-recall regime. It appears that the semi-supervised aspect gives some protection for the method against false positives in the low to mid-recall levels.

### Comparison of Multiple Kernel Learning methods

Next, we compare the different MKL methods ALIGN, ALINGF and p-norm path following on the reconstruction of the set of secretion proteins from the full genome of *S. cerevisiae* when using semi-supervised IOKR as predictor.

**Fig 6:**
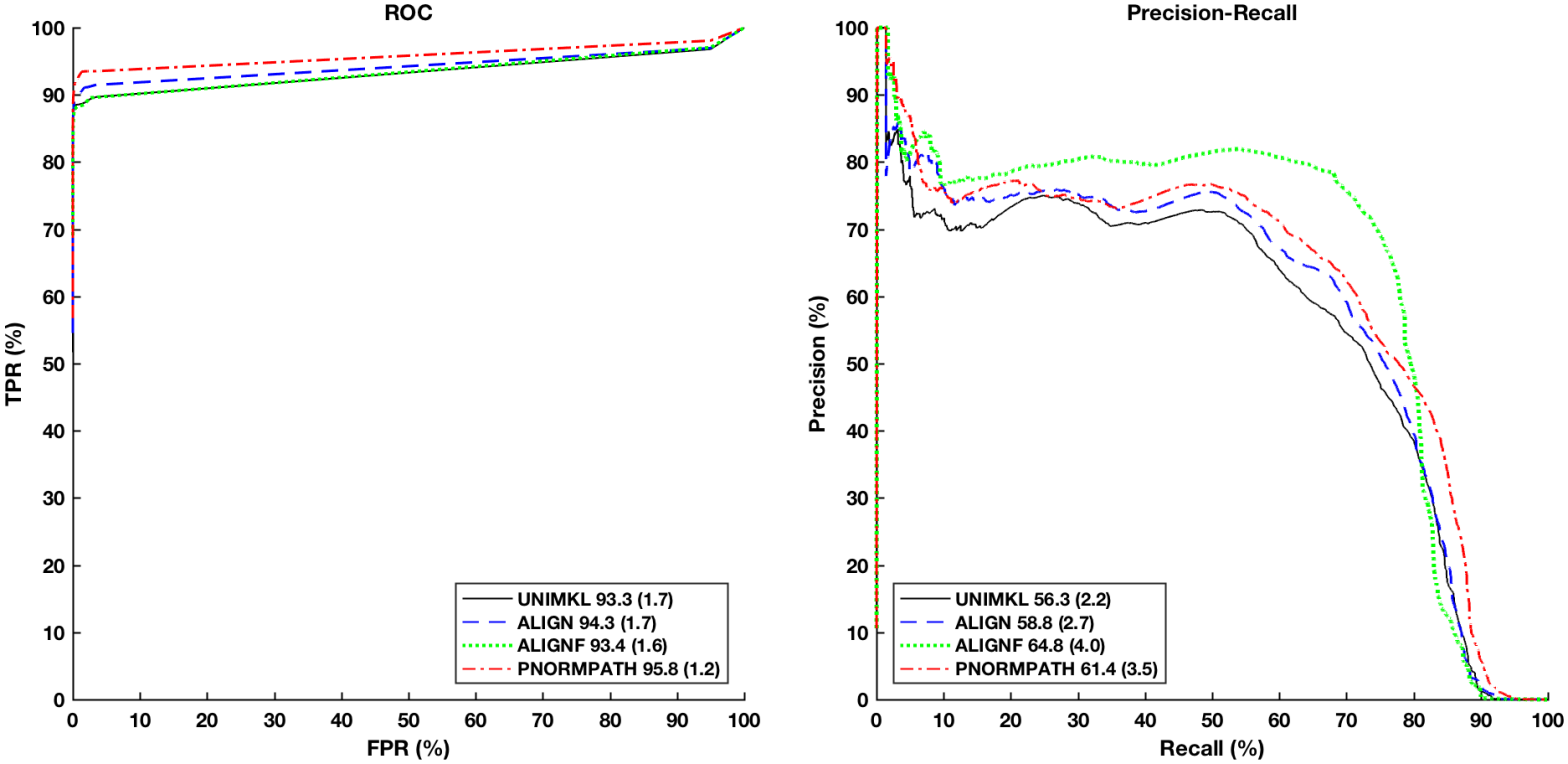
ROC curves and Precision-Recall (PR) curves for predicting secretory PPIs from the full *S. cerevisiae* genome with semi-supervised Input-Output Kernel Regression (IOKR) and different Multiple Kernel Learning (MKL) methods compared to no MKL (UNIMKL). AUCROC and AUPR statistics are shown in the legend for each curve.

The results are shown in Fig 6. It can be seen that the MKL methods perform better than the simple sum of input kernels (UNIMKL) in terms of ROC curve as well as PR curves. Nonetheless, the gains of MKL are smaller than we expected them to be. Looking at the ROC curves, the p-norm path following MKL outperforms the other methods, whereas for the PR measure the simpler ALIGNF outperforms all other methods with p-norm path following being the second best.

### Secretion network prediction for *Trichoderma reesei*

Finally, we evaluate the PPI prediction quality in an inter-species setup, where the training data comes from *S. cerevisiae* and the target species is *T. reesei*. However, no experimental protein interaction data exists for *T. reesei* that could be used as the ground truth. Thus we focus on qualitative analysis of the predicted *T. reesei* secretion network by expert knowledge. For predicting the PPI in *T. reesei* we used semi-supervised IOKR and p-norm path following for learning the input kernel, since this method combination achieved the best performances in the previous experiments.

In order to validate the predicted *T. reesei* secretion network, its genes (*T. reesei* genome version 2.0 [44]) were annotated with a combination of sequence similarity based methods: best BLASTp [45] match to *S. cerevisiae* proteins, best BLASTp match to UniProtKB/Swiss-Prot [46], Interproscan domain predictions [26], PANNZER description line and GO-category predictions [47] and a manually curated set of *Aspergillus niger* protein secretion related genes [48].

**Fig 7:**
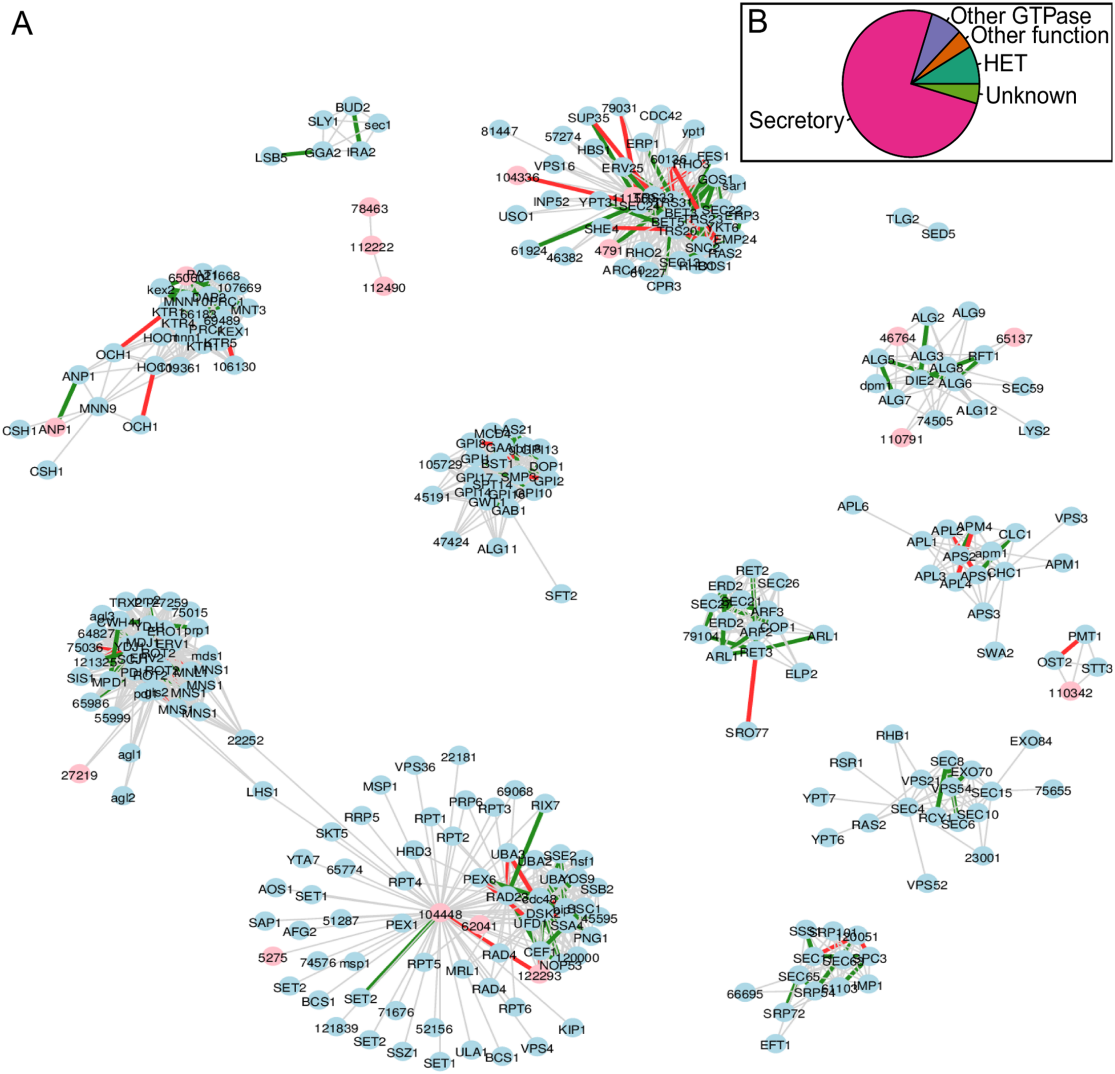
Predicted *T. reesei* secretion network. A) The proteins annotated as secretory (242) and unknown (14) are included. Proteins are nodes and they are labelled with best matching *S. cerevisiae* protein name or if no match was found with *T. reesei* gene ID number. Thick edges signify either negative (red) or positive (green) absolute Pearson correlation of > 0.3 in transcriptomic data. Pink nodes do not have any interactions in STRING. B) Pie chart of functional classes of the 320 proteins included in the *T. reesei* secretion network.

The *T. reesei* secretion network contains in total 320 genes. According to the annotation described above 27 genes belong to the heterokaryon incompatibly family and are sequence wise very similar. This family contains a GTPase domain that could contain similar features as GTPases involved in secretion. 51 genes belong to other than secretion related categories of cellular function. 18 genes were annotated to be related to cell growth, cell wall synthesis and cell motility and six were found to be related to chromatin modification. In general these 24 proteins contain domains related to small molecule modifications of macromolecules such as glycosylation, phosphorylation, ubiquitinylation and methylation. Similar molecular functions are abundant in the known secretion pathway enzymes. 14 of the 51 were annotated as molecular and cellular function unknown (Column ‘Class’ in Table 2). Hence, manual annotation based on sequence similarity suggests a minimum of 75% true positive rate and a maximum of 20% false positive rate. The predicted secretion network, excluding heterokaryon incompatibility family and other than secretion related genes in order to ease visual inspection of know genes, is shown in Fig 7. An alternative layout (S1 Fig) and a table format (S2 Table) of the network are also provided as supplementary material.

**Table 2:** Unknown genes and genes without any interactions in STRING in predicted *T. reesei* secretion network. Column ‘Gene’ contains the *T. reesei* gene ID. ‘In STRING’ tells if the gene has interactions in STRING. Columns ‘Btw’ and ‘Deg’ denote the betweenness and degree network statistics of the corresponding gene. Columns ‘Class’ and ‘Putative secretion pathway component’ are author assigned classifications. ‘Taxon specificity’ gives the largest taxonomic group the gene was found in.

Gene	STRING	Btw	Deg	Class	Protein family or Molecular function	Putative secretion pathway component	Taxon specificity
104448	NO	4075.6	66	Unknown		Protein folding	Pezizomycotina
111569	NO	217.4	43	Unknown		COPII	Pezizomycotina
112222	NO	1.0	2	Unknown	Tetratricopeptide-like helical	?	Pezizomycotina
46764	NO	0.4	7	Unknown		Protein glycosylation	Sordariomycetes
120000	YES	0.2	11	Unknown	Homeodoman superfamily	Protein folding	Trichoderma
105729	YES	0.0	11	Unknown		GPI-biosynthesis	Trichoderma
122293	NO	0.0	9	Unknown		Protein folding	Pezizomycotina
4791	NO	0.0	7	Unknown		COPII	Pezizomycotina
62041	NO	0.0	5	Unknown		Protein folding	Pezizomycotina
110342	YES	0.0	3	Unknown		Protein glycosylation	Pezizomycotina
104336	NO	0.0	2	Unknown	SnoaL-like domain	COPII	Trichoderma
110791	NO	0.0	2	Unknown		Protein glycosylation	Trichoderma
112490	NO	0.0	1	Unknown		?	Pezizomycotina
47424	YES	0.0	6	Unknown		GPI-biosynthesis	Sordariomycetes
65060	NO	0.0	17	Secretion	Golgi mannosyltransferase complex subunit	Golgi processing	Pezizomycotina
81211/ANP1	NO	0.0	2	Secretion	Golgi mannosyltransferase complex subunit	Golgi processing	Fungi
78463	NO	0.0	1	Regulatory functions	TPR repeat protein		Pezizomycotina
5275	NO	0.0	1	Chromatin modification			Fungi
27219	NO	0.0	2	Cell growth, wall, motility	Alpha-galactosidase		Trichoderma
65137	NO	0.0	2	Cell growth, wall, motility	Endo-1,3-beta-glucanase		Pezizomycotina

16 of the 320 genes were found to have no interactions in the protein-protein interaction database STRING [4] (Column ‘In STRING’ in Table 2). For 2 of these we found strong similarity based evidence that they are part of the Golgi mannosyltransferase complex and could be considered as false negative predictions by STRING.

For each gene annotated as unknown, a putative role in the secretion pathway machinery was assigned based on their position in the predicted network (Column ‘Putative secretion pathway component’ in Table 2). To estimate the novelty of such secretion pathway components the taxonomic distribution of the unknown genes was estimated with multi-genome protein clustering [49] (Column ‘Taxon Specificity’ in Table 2). All unknown genes were found to be restricted to the subphylum Pezizomycotina or a smaller taxon with-in Pezizomycotina.

In order to further validate the *T. reesei* secretion network we used a combined transcriptomics data set of public and in-house data (see Methods). Pearson correlation of the expression values of all gene pairs that have a predicted PPI (an edge in the PPI network) was computed. The average of absolute values of these correlations was found to be 0.2 with an empirical p-value of p < 0.05. This p-value was calculated by rewiring the network 1000 times with the igraph function ‘rewire’ [50] and counting the average of absolute correlation each time. Absolute correlations above 0.3 are highlighted in Fig 7.

## Discussion

Experimental measurement of protein-protein interactions is technically demanding and often different methods can give conflicting results [7, 51]. Also, even a reliably measured interaction might not have a detectable biological function. To circumvent such challenges we use an expert curated interaction network of functional associations derived from numerous experiments [24].

We tested several recent machine learning methods for the task of PPI prediction. Classification models tested included pairwise kernels, output kernel trees as well as supervised and semi-supervised input-output kernel regression. The methods differed in performance depending on whether ROC or PR was used as the evaluation metric. Semi-supervised IOKR proved to be the best compromise when both evaluation metrics were taken into account: it had the best PR performance and a reasonable ROC—this choice puts an emphasis on good performance in the positive class, required for reliable network reconstruction.

Multiple kernel learning methods tested included uniform kernel combination, methods based on centered kernel alignment as well as the newly proposed p-norm path following algorithm. In our tests, we found that generally p-norm path following performed best in the ROC metric while other methods were close to each other in performance. In the PR metric, ALIGNF outperformed the other methods and p-norm path following being second best. Altogether, p-norm path following seems to give the best performance, although the improvements of MKL over no MKL were smaller than expected.

To demonstrate our prediction approach we predict protein secretion network for *T. reesei*, an industrially important protein production organism, which has no experimentally verified PPIs to date. Novel understanding of their protein secretion network machinery could have significant impact in the generation of improved protein production strains through targeted engineering.

For *T. reesei* we find that the predicted network is well supported by sequence similarity based manual annotation and by transcriptomics data. Most importantly the predicted network includes 14 previously unknown genes that are taxonomically restricted to Pezizomycotina and hence could explain their exceptional protein secretion capabilities.

Finally we note that our set up does not need complex external database systems or specialized experimental data to be generated, but relies on data available through standard sequence searches, evaluated through fast machine learning models. Hence, our methods are amenable to local implementation as part of a genome annotation pipeline.

## Supporting Information

S1 Table Protein feature data sources. Protein feature data sources used in training the PPI prediction models.
(XLSX)

S2 Table Trichoderma reesei secretion network. Predicted protein-protein interactions of Trichoderma reesei secretion pathway.
(XLSX)

S1 Fig Alternative layout of predicted *T. reesei* secretion network. In this layout interactions of individual genes are easier to inspect with the cost of less clear overall structure.
(TIFF)
