# Supplementary figures and images for: Machine Learning of Protein Interactions in Fungal Secretory Pathways
Source: PLoS One. 2016 Jul 21;11(7):e0159302. doi: 10.1371/journal.pone.0159302 (PMC4956264; doi:10.1371/journal.pone.0159302)

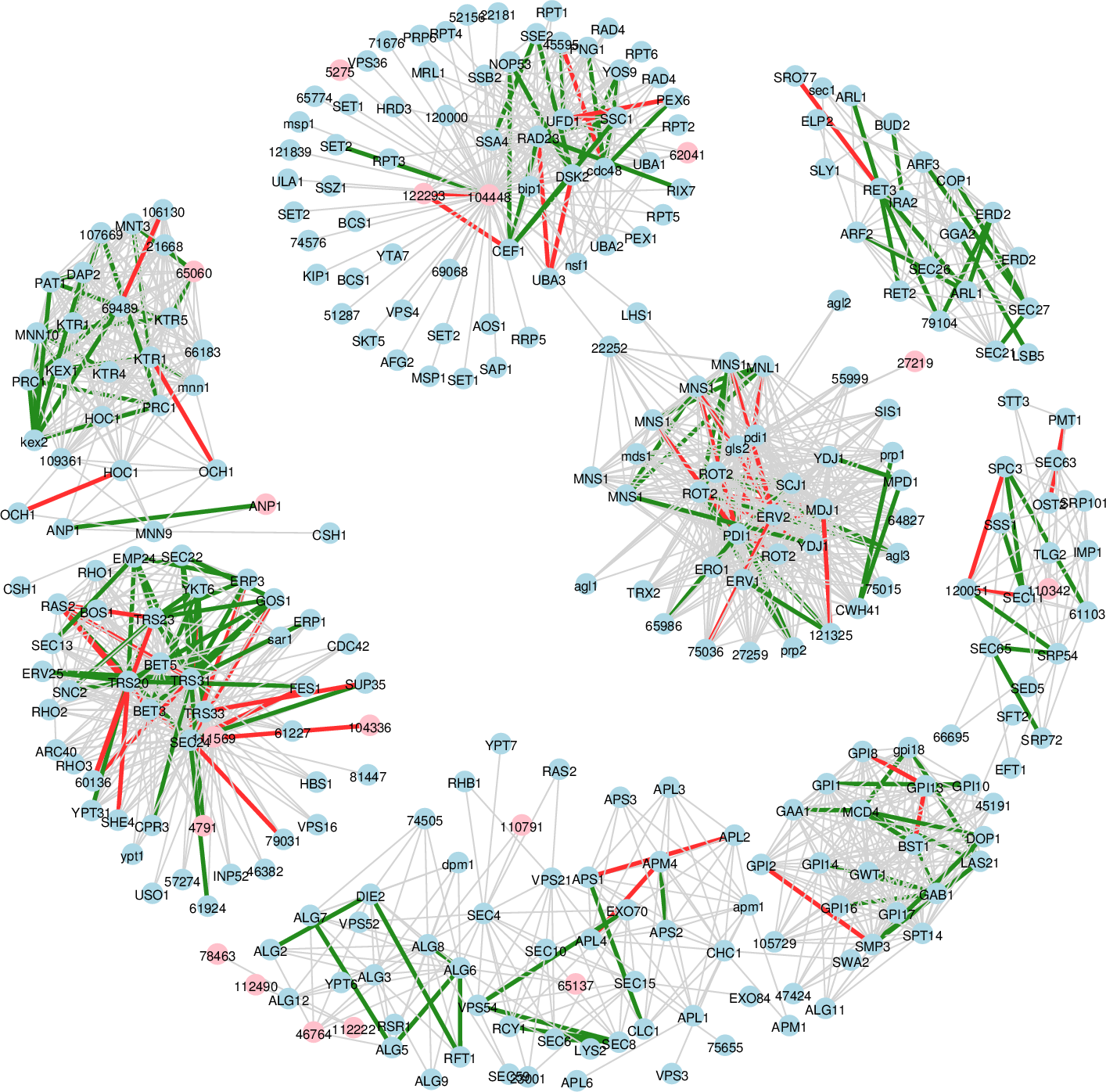

Supplement: S1 Fig — In this layout interactions of individual genes are easier to inspect with the cost of less clear overall structure. (TIFF) [file pone.0159302.s003.tiff]
